# The global challenge of *Candida auris* in the intensive care unit

**DOI:** 10.1186/s13054-019-2449-y

**Published:** 2019-05-02

**Authors:** Andrea Cortegiani, Giovanni Misseri, Antonino Giarratano, Matteo Bassetti, David Eyre

**Affiliations:** 1 Department of Surgical, Oncological and Oral Science (Di.Chir.On.S.). Section of Anesthesia, Analgesia, Intensive Care and Emergency. Policlinico Paolo Giaccone, University of Palermo, Via del vespro 129, 90127 Palermo, Italy; 2 Infectious Diseases Division, Department of Medicine, University of Udine and Santa Maria della Misericordia University Hospital, Piazzale Santa Maria della Misericordia 15, Udine, Italy; 3 Oxford University Hospitals NHS Foundation Trust, Oxford, OX3 9DU UK

Since the first isolation of *Candida auris* in 2009, scientific community has witnessed an exponential emergence of infection episodes and outbreaks in different world regions [1]. According to the Centers for Disease Control and Prevention (CDC), 560 cases of *C. auris* infections have been notified in the United States as 31 January 2019. It is likely that many cases are missed, due to its misidentification with other non-*albicans Candida* spp. (e.g., *C. haemulonii*) by common microbiological diagnostic methods (https://www.cdc.gov/fungal/diseases/candidiasis/tracking-c-auris.html). Most of the reports occurred in critically ill adults, with risk factors for invasive fungal infections, such as immunosuppression, surgery, or indwelling catheters. The most common form of infection was candidemia, with a crude mortality of nearly 30%, but up to 70% in some reports [2].

Despite implementation of countermeasures to limit colonization and infections in intensive care units (ICUs), cases continue to be reported, with a tendency to an endemic pattern [3]. This reflects the ability of *C. auris* to persist in clinical environment, facilitating its transmission within critical care setting. Multidrug-resistant (MDR) pattern and has been frequently observed (around 40%) with serious and complex consequences for antifungal therapy [4].

In view of *C. auris* progressive spread and treatment concerns, attention should be focused on the following A.U.R.I.S. major issues (Fig. 1):

1. Worldwide Alert
2. AntifUngal treatment resistance
3. Resilience and mechanisms of transmission
4. Implementation of infection prevention and control measures
5. Surveillance

**Fig. 1:**
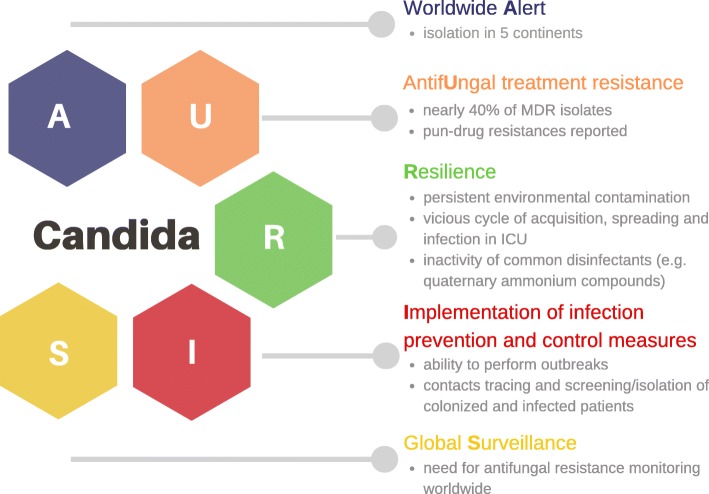
Major issues related to *Candida auris.* Major issues related to *Candida auris* described with A.U.R.I.S. outline. MDR, multidrug resistant; ICU, intensive care unit

## Worldwide Alert

Following the first isolation in Japan, cases have been reported in several countries in five continents. Although uncommon for fungi, *C. auris* has the ability to cause outbreaks, as seen in India, the UK, Spain, the USA, Venezuela, Colombia, and South Africa [1]. It is still debated whether *C. auris* emerged in one region with subsequent spreading to others, or if it emerged independently across different countries. Evidence from genomic sequencing demonstrates different clades of *C. auris* show strong geographic structure, with independent emergence in East and South Asia, Africa, and South America [1, 5, 6].

## AntifUngal treatment resistance

To date, there are not established minimum inhibitory concentrations (MICs) breakpoints for susceptibility testing of *C. auris*. Antifungal susceptibility data from three continents demonstrated that nearly 40% were MDR, with strains being resistant to fluconazole (90%), amphotericin B (30–40%) and echinocandins (5–10%). Moreover, a small percentage were also resistant to all antifungals actually available [4, 6]. *C. auris* demonstrates a high propensity to develop antifungal resistance under selective pressure. Recent studies demonstrated mutations in *ERG*11 (encoding lanosterol demethylase, the target of azoles) and *FKS*1 genes (encoding 1,3-beta-glucan synthase, the target of echinocandins) [1, 7].

The recommended antifungals for *C. auris* treatment are mainly based on in vitro testing and on the most frequently retrieved resistance profiles. Echinocandins are the recommended first-line treatment, pending specific susceptibility testing. Lipid formulation of amphotericin B should be an alternative in patients not responding to echinocandins. Close monitoring to early detect therapeutic failures and evolution of antifungal resistance is needed. New antifungals (e.g., SCY-078, APX001A/APX001, and rezafungin) have been tested with success but they are not available to date for clinical use [1].

## Resilience and mechanisms of transmission

Unlike others *Candida* species, *C*. *auris* can colonize different anatomical sites (e.g., skin, skin, rectum, axilla, stool) and contaminate hospital equipment and surfaces, creating a *vicious cycle* of acquisition, spreading, and infection, particularly in ICUs. Indeed, bed, chairs, and monitoring tools (e.g., pulse oximeters, temperature probes) were contaminated during outbreaks [8]. Recently, Eyre et al. [9] published the results of a patients’ and hospital environmental screening program in Oxford, UK, after 70 patients (66 admitted to a neuro-ICU) were identified as being colonized or infected by *C. auris*. Seven patients developed an invasive infection during hospital stay. *C*. *auris* was detected mainly on skin-surface axillary temperature probes and other reusable tools. In patients monitored with skin-surface temperature probes, the risk of *C. auris* infection/colonization was seven times higher. Adoption of specific bundles of infection control had no significant effects until removal of the temperature probes [9].

Recent studies have confirmed that *C. auris* can form biofilms, with a high variation of capacity of production depending on the *C. auris* strain considered [10]. Biofilm may present reduced susceptibility to hydrogen peroxide and chlorhexidine [11].

Quaternary ammonium compounds and cationic surface-active products seem to be ineffective against *C. auris*. Chlorine-based products appear to be the most effective for environmental surface disinfection [12]. Chlorine-based disinfectants (at a concentration of 1000 ppm), hydrogen- peroxide, or other disinfectants with documented fungicidal activity are recommended for environmental cleaning by the European CDC (ECDC) [13].

## Implementation of infection prevention and control measures

CDC and ECDC released recommendations for *C. auris* case and outbreak management [13]. Usually, outbreaks follow an exponential increase in the number of affected patients. It is mandatory to trace contacts with the aim to achieve early identification and screening of possible colonized patients that might be responsible for persistence of *C. auris*. Patients potentially or already colonized should be placed in single rooms with contact isolation precautions. Screening should be applied for contacts and patients previously hospitalized in healthcare settings where *C. auris* isolation was confirmed. Hand hygiene (with alcohol or chlorhexidine hand rubs), wearing of protective clothing, and skin and environmental/equipment decontamination should be performed to prevent ongoing transmission.

## Global Surveillance

Aiming to support implementation measures on global surveillance on antimicrobial resistances, in 2016, the World Health Organization [14] launched the Global Resistance Surveillance System (GLASS). The emergence of *C. auris* and progressive spread of infections caused by other resistant pathogens has strengthened the need for a surveillance network for antimicrobial resistance globally for critically ill patients’ safety.

It is hard to predict future *C. auris* diffusion. There will be outbreaks also in countries in which *C. auris* has been not reported yet? Will new MDR clones continue to emerge? Will we be able to apply effective antifungal stewardship programs and control measures? By now, global surveillance, improving knowledge, and taking care of the A.U.R.I.S. major issues may be the best ways to face *C. auris* challenge.

## Abbreviations

CDC: Centers for Disease Control and Prevention
ECDC: European Center for Disease Prevention and control
GLASS: Global Resistance Surveillance System
ICU: Intensive care unit
MDR: Multidrug resistant
MIC: Minimum inhibitory concentrations
